# Anticancer Effects of *Helminthostachys zeylanica* Ethyl acetate Extracts on Human Gastric Cancer Cells through Downregulation of the TNF-α-activated COX-2-cPLA2-PGE_2_ Pathway

**DOI:** 10.7150/jca.64638

**Published:** 2021-10-17

**Authors:** Ming-Ming Tsai, Horng-Chyuan Lin, Ming-Chin Yu, Wan-Jung Lin, Mei-Yi Chu, Ching-Ching Tsai, Ching-Yi Cheng

**Affiliations:** 1 Department of Nursing, Division of Basic Medical Sciences, Research Center for Chinese Herbal Medicine, College of Human Ecology, Chang Gung University of Science and Technology, Taoyuan, Taiwan and Department of General Surgery, Chang Gung Memorial Hospital at Chiayi, Chiayi, Taiwan.; 2 Department of Thoracic Medicine, Chang Gung Memorial Hospital at Linkou and College of Medicine, Chang Gung University, Taoyuan, Taiwan.; 3 Department of Surgery, New Taipei Municipal TuCheng Hospital, Chang Gung Memorial Hospital at Linkou, and College of Medicine, Chang Gung University, Taoyuan, Taiwan.; 4 Graduate Institute of Health Industry Technology, Research Center for Chinese Herbal Medicine and Research Center for Food and Cosmetic Safety, College of Human Ecology, Chang Gung University of Science and Technology, Taoyuan, Taiwan.; 5 Department of Nursing, College of Nursing, Chang Gung University of Science and Technology, and Department of Cardiology, Chang Gung Memorial Hospital at Linkou, Taoyuan, Taiwan.; 6 Department of Pulmonary Infection and Immunology, Chang Gung Memorial Hospital at Linkou, Taoyuan, Taiwan.

**Keywords:** *Helminthostachys zeylanica*, anti-inflammation, anticancer, gastric cancer

## Abstract

**Background:** Gastric cancer (GC) is the second most prevalent cancer worldwide and the eighth most common cause of tumor-related death in Taiwan. *Helminthostachys zeylanica*, a flavonoid compound, has anti-inflammatory, immunomodulatory, and anticancer effects. We examined whether an extract of *H. zeylanica* (E1 and E2) has potential as a treatment for GC.

**Methods:** We investigated the effects (pro-apoptosis, pro-autophagy, and antiproliferation ability) of *H. zeylanica*-E2 on cell viability in healthy gastric epithelial (GES-1) and GC cells (AGS and BGC823). *H. zeylanica*-E2 was toxic to GC cells but had little or no toxicity to normal cells.

**Results:** In this study, *H. zeylanica*-E2 induced apoptosis through caspase 3/7, Bcl-2, Bax, cyclooxygenase-2 (COX-2), and cleaved poly (ADP-ribose) polymerase pathways in GC cells. In addition, it increased autophagy by stimulating autophagy-related protein (ATG)5, ATG7, LC3-I/LC3-II, and inhibiting COX-2 activity in GC cells. We also found that *H. zeylanica*-E2 exhibited antiproliferation ability through cell cycle arrest in G0/G1 and G2/M and suppressed the migration of GC cells. The anticancer effects of *H. zeylanica*-E2 in GC cells might be mediated partly through inhibition of tumor necrosis factor-α (TNF-α)-activated proinflammatory cytosolic phospholipase A2 (cPLA2)-COX-2-prostaglandin E_2_ (PGE_2_) pathway.

**Conclusions:** Our results suggest that *H. zeylanica*-E2 has potential as a novel adjunctive agent for the treatment of GC.

## Introduction

Gastric cancer (GC) has a high incidence worldwide and its high mortality rate is related to malignant tumors 1. According to the 2019 cancer statistics from the Department of Health in Taiwan, GC is the eighth leading cause of cancer-associated mortality in Taiwan (https://www.mohw.gov.tw/cp-16-54482-1.html). GC is rarely detected at an early stage because it often causes no clinical symptoms and it is too advanced for curative surgical resection in more than 30% of patients with GC 2-4. The 5-year survival outcome for patients with late-stage GC is poor after the initial diagnosis 5. At present, gastrectomy remains the main therapy for GC 4, 6-8. Therefore, the discovery of novel medicines to help the development of therapeutic strategies for GC management is necessary 9. Chemotherapy is used for patients with advanced or recurrent GC and includes anti-vascular endothelial growth factor (anti-VEGF), anti-VEGF receptor, tyrosine kinase inhibitors, and Her2/Neu (also known as ErbB-2)-mediated pathway blockers 10, 11. Traditional herbal medicines are also used in combination with chemotherapy for the treatment of lung cancer, hepatocellular carcinoma (HCC), and GC 12-18.

The inflammatory response is one of the host defenses against tumors and can lead to tumor destruction with favorable prognosis. However, inflammation can also be related to poor clinical prognosis in patients with GC 19. Chronic infection of the gastric mucosa with *Helicobacter pylori* is recognized as an important risk factor for GC 20. Infection-induced chronic inflammation associated with inflammatory protein expression plays an important role in GC progression 21, 22. Masami *et al*. found that *H. pylori* infection can promote the secretion of TNF-α and related inflammatory proteins, such as COX-2, PGE_2_, and cPLA2 through the nuclear factor κB (NF-κB) pathway 23-25. Epstein-Barr virus is involved in almost 10% of all GC cases 26. Chronic inflammation leads to oxidative stress in immune cells and gastric epithelial (GES-1) cells, which secrete inflammatory factors such as proinflammatory cytokines and chemokines. These inflammatory factors are the main contributor to DNA damage, apoptosis that can lead to gastric ulcers, and even GC. Failure of the host to eradicate infection with *H. pylori* or other viruses can cause chronic inflammation.

New Chinese herbal medicines with anti-inflammatory and antioxidative activities may be helpful in the prevention of chronic gastritis or as adjuvant chemotherapy for GC and may increase the survival rate of patients with GC 27-29. *Helminthostachys zeylanica* is a herbaceous plant widely distributed in Southeast Asia that is rich in flavonoid compounds such as ugonins A-U 30-33. Previous studies have found that neouginin A isolated from *H. zeylanica* can reduce the lipopolysaccharide (LPS)-induced expression of inflammation-related proteins through the NF-κB signaling pathway in macrophages 34. Ugonin M improves recovery from LPS-induced acute lung injury by regulating the NF-κB and mitogen-activated protein kinase (MAPK) signaling pathways 35. *H. zeylanica* has extensive pharmacological effects including anti-inflammatory, antioxidative, antiosteoporosis, hepatoprotective, immunomodulatory, and neuroprotective activities 30-32, 36. Other researchers have reported that *H. zeylanica* extracts reduce inflammation and matrix metallopeptidase 9 (MMP-9) expression in bradykinin-activated brain astrocytes 36. *H. zeylanica* extracts are also used to treat fever, inflammatory diseases, and bone loss caused by osteoarthritis in Taiwan 30, 31, 37, 38. However, it is unknown whether the anticancer effects of *H. zeylanica* may be useful for the treatment of GC. We examined whether *H. zeylanica* extracts have anticancer effects in GC cells and, if so, we tried to identify the underlying mechanisms.

## Methods

### The preparation of *H. zeylanica* extracts

The roots of *H. zeylanica* were purchased in Wanhua, Taiwan, and identified by Mr. Jun-Chih Ou. The roots of *H. zeylanica* were extracted initially with 50% ethanol. Then, the concentrated ethanol extract was separated by Sephadex LH-20 gel column chromatography eluting to get *H. zeylanica*-E1 (H_2_O extract) and *H. zeylanica*-E2 (EtOAc extract). The detailed preparation of *H. zeylanica*-E1 and E2 as described in previous literature 36 and 39. The *H. zeylanica*-E1 and E2 in this article were prepared in keeping with a previously reported protocol 36, 39.

### Cell Lines and Cell Culture

The human GC cell line, AGS, was obtained from the American Type Culture Collection (ATCC, CRL-1739). GES-1 and BGC823 cells were obtained from Dr. Q.X. Chen and Dr. D.W. Zhou, Xiamen University, Fujian, China. All cells were cultured in RPMI 1640 medium (Invitrogen, Carlsbad, CA, USA) containing 10% fetal bovine serum (FBS) plus 100 IU/mL penicillin G, 100 mg/mL streptomycin sulfate (Sigma-Aldrich, St Louis, MO, USA), and nonessential amino acids at 37 °C in 95% air and 5% CO_2_ as described previously 40. The cells were either left untreated or were treated with *H. zeylanica*-E2 and/or TNF-α.

### MTT Cell Survival Assay

GES-1, AGS, and BGC823 cells (5000 cells) were seeded into 96-well plates with serum-free RPMI 1640 medium and cultured for 24 h at 37 °C. The cells were treated with *H. zeylanica-*E1 (0, 10, 50, 100, 500, 1,000, 5000, or 10 000 μg/mL) or *H. zeylanica*-E2 (0, 1, 5, 10, 20, 40, 80, or 100 μg/mL) 39 for 24 or 48 h. Next, 0.25 mg/mL MTT reagent (Bio-Rad Laboratories, Hercules, CA, USA) was added (100 μL/well), the incubation was continued for 0.5 h, and 100 μL of dimethyl sulfoxide was then added to the medium. The optical density at 570 nm was detected using a microplate reader (SpectraMax i3; Kelowna International Scientific Inc., CA, USA) as described previously 36, 40.

### Quantitative Reverse Transcription Polymerase Chain Reaction

GC cells were cultured in serum-free RPMI medium in 9 cm dishes for 24 h at 37 °C and then treated with* H. zeylanica-*E2 or a selective COX-2 inhibitor (celecoxib, CLC) for 0, 24, or 48 h. Total RNA was extracted using TRIzol® (Invitrogen), and the concentration of all RNA samples was detected using a Nano-100 Micro-Spectrophotometer (CLUBIO, Taipei, Taiwan) 41. Next, cDNA was synthesized using an iScript™ cDNA Synthesis Kit (Bio-Rad Laboratories) and amplified on a spectrofluorometric thermal cycler (iCycler; Bio-Rad Laboratories). To assess* COX-2* mRNA expression in GC cells, qRT-PCR was performed as described previously, using *ACTB* as the internal control 42, 43. Fluorescence emitted by SoFast EvaGreen Supermix was assayed using the CFX Connect Real-Time System (both from Bio-Rad). The primers used for RT-qPCR were as follows: human *PTGS2* (forward, 5′-CTGCGCCTTTTCAAGGATGG-3′, and reverse, 5′-CCCCACAGCAAACCGTAGAT-3′) and human *ACTB* (forward, 5′-AAAGACCTGTACGCCAACAC-3′, and reverse, 5′-GTCATACTCCTGCTTGCTGAT-3′). Relative gene expression was determined by the ΔΔCt method, where Ct represents the mean threshold cycle.

### Western Blot

Western blotting was conducted as described previously 43. Total proteins were extracted from GC cells grown in serum-free RPMI medium and seeded in 9 cm dishes and cultured for 24 h at 37 °C. The cells were then treated with *H. zeylanica-*E2 or CLC plus TNF-α or the arachidonic acid (AA) derivative AACOCF3, which is a cPLA2 inhibitor for 0, 24, or 48 h. Next, the cells were harvested and the proteins were separated by sodium dodecyl sulfate-polyacrylamide gel electrophoresis and then transferred to polyvinylidene fluoride membranes. Western blotting was completed using standard procedures, as described previously 44. Rabbit anti-Bcl-2, anti-Bax, anti-cleaved PARP, anti-COX-2, anti-autophagy-related protein (ATG)5, anti-ATG7, anti-Beclin-1, and anti-LC3-I/LC3-II were purchased from Cell Signaling Technology (Danvers, MA, USA) and used at a dilution of 1:1000. Mouse anti-GAPDH antibody was obtained from Santa Cruz Biotechnology (Santa Cruz, CA, USA). Finally, the protein bands were detected using an ECL-detecting reagent (Visual Protein Biotech, New Taipei City, Taiwan) and captured using a UVP BioSpectrum 500 Imaging System (UVP, Upland, CA, USA). Image densitometry analysis was quantified using Image Lab™ Software (Bio-Rad Laboratories).

### ELISA

After culture for 24 h, GC cells were incubated with serum-free RPMI 1640 medium. PGE_2_ protein level in the medium from cells treated with *H. zeylanica-*E2 or CLC for 0, 24, or 48 h was measured using a human protein PGE_2_ enzyme immunoassay kit (Enzo Life Sciences, Farmingdale, NY, USA). Fluorescence intensity was measured at 450 nm using a SpectraMax i3 microplate reader (Kelowna International Scientific Inc.) 40, 45.

### Flow Cytometry Analysis of Apoptosis Using Annexin V-Propidium Iodine Staining and Caspase 3/7 Staining Assays

GC cells were seeded in 9 cm dishes and grown in serum-free RPMI 1640 medium for 24 h at 37 °C and then treated with 10 or 5 μg/mL *H. zeylanica*-E2 for 0, 24, or 48 h. Cell apoptosis was assessed using flow cytometry. Cells were harvested using standard procedures as described previously 46 and prepared using a Muse® Annexin V & Dead Cell Kit and Muse® Caspase-3/7 Assay Kit. Data were collected and analyzed using a Muse® Cell Analyzer flow cytometer (all kits and equipment from Merck).

### Cell Cycle Assay

GC cells were treated as described above for the cell cultures used for cell cycle analysis by flow cytometry and harvested using standard procedures as described previously 47. The cells were prepared using a Muse® Cell cycle Kit, and the data were collected and analyzed using a Muse® Cell Analyzer flow cytometer (all from Merck).

### Colony-Forming and Cell Proliferation Assays

In the colony-forming assay, 1×10^3^ GC cells were seeded in serum-free RPMI 1640 medium in six-well plates and grown for 24 h. GC cells were treated with 0, 10, or 5 μg/mL *H. zeylanica*-E2. After incubation for 2 weeks, colonies were fixed and stained with crystal violet. Colony numbers were photographed and measured using Image Lab™ Software (Bio-Rad) 47. For the analysis of cell proliferation, 1×10^3^ GC cells were plated in wells of a six-well plate and treated as described for cell culture. Viable cells were trypsinized and counted on days 1, 7, 9, and 11 or 13.

### Scratch Wound-Healing Assay

The *in vitro* scratch wound-healing assay was used to assess the effects of *H. zeylanica*-E2 on the metastatic activity of GC cells. GC cells (3 × 10^5^) in serum-free RPMI 1640 medium were seeded in a six-well plate and incubated for 24 h. GC cells were treated with 10 or 5 μg/mL *H. zeylanica*-E2 and 10 μM hydroxyurea for 0, 24, or 48 h. When the cells had reached 100% confluence, two parallel wounds of 1 mm were made using a pipette tip. The number of cells that moved into the wound was measured after 48 h and photographed using a Leica Microsystems microscope (Wetzlar, Mannheim, Germany) as described previously 48.

### Statistical Analysis

Quantitative data were analyzed using Student's-*t*-test (for two groups) or one-way ANOVA (for more than two groups). Values are expressed as mean ± SEM. A *p* value < 0.05 was considered significant 49. All experiments were performed at least three times independently. The data from Western blotting were analyzed using image Lab™ 5.0 software (Bio-Rad Laboratories). All results were analyzed using GraphPad Prism software (GraphPad, San Diego, CA, USA).

## Results

### Effects of H. zeylanica Extracts (E1 and E2) on the Viability of GC and Normal Cells

The compounds in *H. zeylanica* extracts (E1 and E2) were analyzed using high-performance liquid chromatography (HPLC). The *H. zeylanica* secondary extracts with *H. zeylanica*-E1 and *H. zeylanica*-E2 were prepared. We found that both *H. zeylanica* extracts contained mostly flavonoids compounds (the major compounds are ugonins J and K) 39.

**Figure 1:**
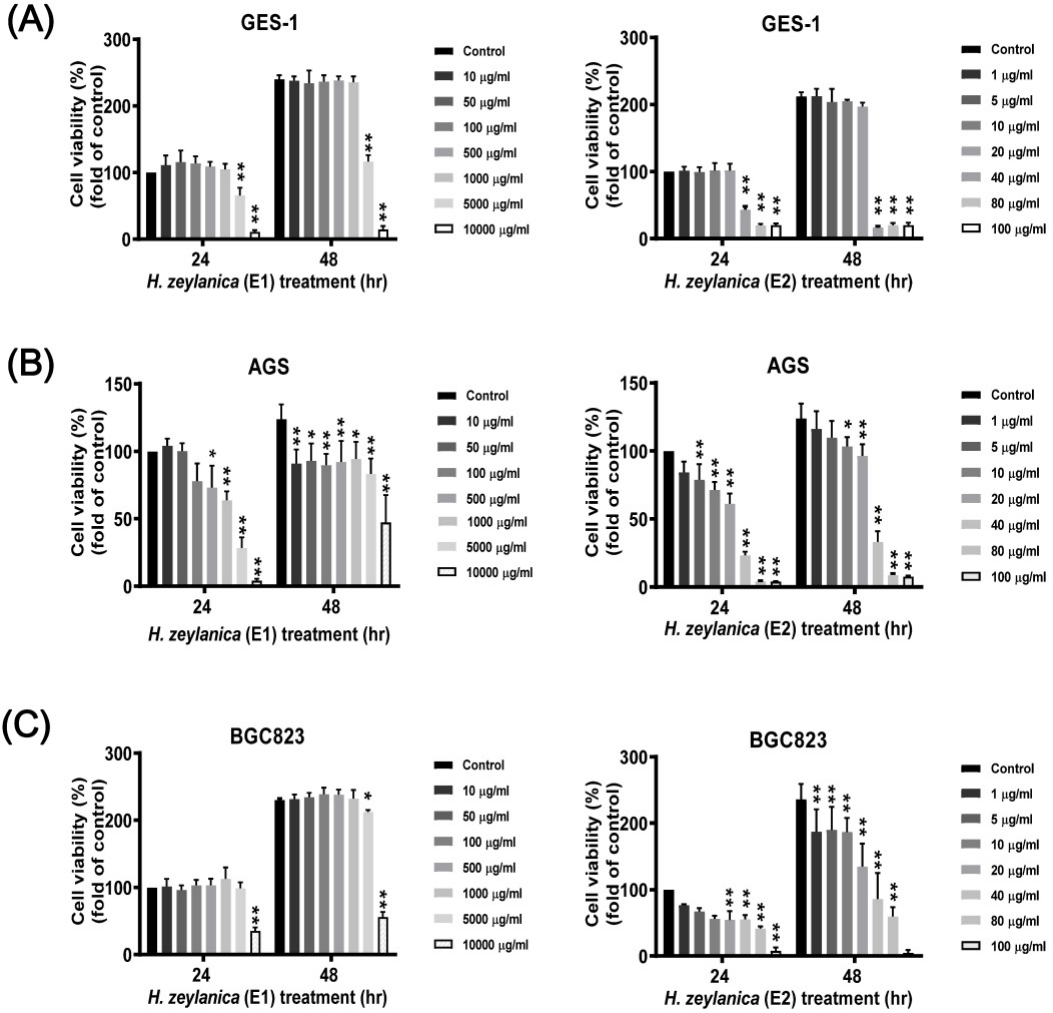
** Effects of* H. zeylanica* extracts (E1 and E2) on the viability of GC and normal GES-1 cells.** (**A**) normal GES-1 cell line, (**B**) AGS, and (**C**) BGC823 cells were treated with *H. zeylanica*-E1 and *H. zeylanica*-E2 at the concentrations indicated for 24 or 48 h. Viability of cells exposed to various concentrations of *H. zeylanica* extracts (E1 and E2) was assessed using the MTT assay and were expressed relative to that of untreated cells incubated for 24 h. Data are presented as mean ± standard error of the mean (SEM) for three independent experiments. Student's-*t*-test or one-way analysis of variance (ANOVA) was used for comparison with the control values. (**p < 0.01, *p < 0.05).

Next, to evaluate whether the *H. zeylanica* extracts E1 and E2 exhibited anticancer effects *in vitro*, serial dilutions of these extracts were prepared in RPMI medium and incubated with GC and normal GES-1 cells for 24 or 48 h. 3-[4,5-dimethylthiazol-2-yl]-2,5 diphenyl tetrazolium bromide (MTT) assay was used to assess the effects of the two extracts on cell viability in two GC cell lines (AGS and BGC823) and the normal GES-1 cell line. *H. zeylanica*-E1 treatment up to the concentration of 1000 μg/mL for 24 or 48 h did not affect the viability of GES-1 cells (Figure 1A, left). By contrast, these concentrations significantly altered the viability of AGS cells incubated for 24 h and 48 h (Figure 1B, left). Treatment with E1 did not alter the viability of BGC823 cells at most concentrations and for both incubation times, except at a concentration of 10000 μg/mL (Figure 1C, left). These results indicated that *H. zeylanica*-E1 up to the concentration of 1000 μg/mL was therefore not suitable for studying different types of GC cells. This extract was not used in the follow-up experiments. *H. zeylanica*-E2 up to the dose of 20 μg/mL for 24 h and 48 h did not reduce the viability of GES-1 cells (Figure 1A, right) but had a significant effect on the viability of AGS cells (Figure 1B, right) and BGC823 cells (Figure 1C, right) in the concentration-dependent manner. These cell viability profiles showed that *H. zeylanica*-E2 induced dose- and time-dependent effects on the death of GC cells. Our results suggest that *H. zeylanica*-E2 is selectively toxic to GC cells, but has minimal or no toxicity for normal GES-1 cells (Figure 1). Therefore, we chose to use *H. zeylanica*-E2 at concentrations of 10 μg/mL in AGS cells and 5 μg/mL in BGC823 cells for further evaluation of the anticancer effects.

### Effects of H. zeylanica-E2 on GC Cell Apoptosis

**Figure 2:**
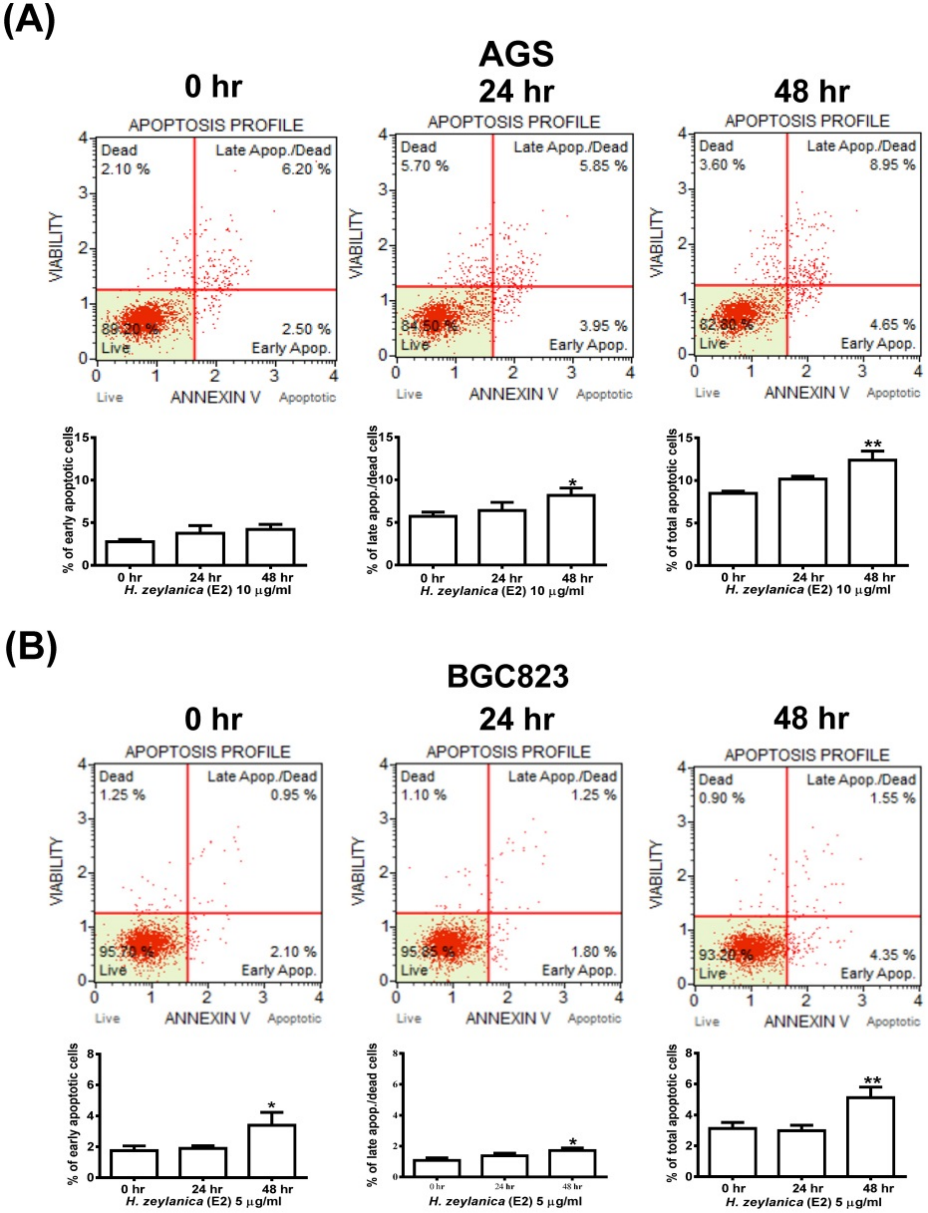
** Effects of *H. zeylanica*-E2 on GC cell apoptosis.** (**A**) AGS cells were treated with *H. zeylanica*-E2 (10 μg/mL), and (**B**) BGC823 cells were treated with *H. zeylanica*-E2 (5 μg/mL) for 0, 24, or 48 h. The cells from different experimental groups were collected and incubated with Muse™ Annexin V & Dead Cell reagent (Merck Millipore, Billerica, MA, USA) for 20 min at room temperature and then analyzed using a Muse^TM^ Cell Analyzer (Merck). Four populations of cells (live, early apoptotic, late apoptotic, and dead) were quantified. The upper left shows mostly nuclear debris: Annexin V^-^ and 7-AAD^+^; lower left shows nonapoptotic cells: Annexin V^-^ and 7-AAD^-^; lower right shows early apoptotic cells: Annexin V^+^ and 7-AAD^-^; and the upper right shows late-stage apoptotic and dead cells: Annexin V^+^ and 7-AAD^+^. The figures below (A, B) are the quantitative results. Data are presented as mean ± SEM of at least three independent experiments. Student's-*t*-test or one-way ANOVA was used for comparison with the control values. (**p < 0.01, *p < 0.05).

We next explored whether the molecular mechanism responsible for the *H. zeylanica*-E2 inhibition of the growth of GC cells involves apoptosis. Cells were stained with Annexin V-7-amino actinomycin (7-AAD) and analyzed by flow cytometry (Figure 2A). AGS cells treated with *H. zeylanica*-E2 exhibited apoptosis in a time-dependent manner, especially in late-stage apoptosis. *H. zeylanica*-E2 treatment significantly increased the total cell apoptotic rate from 8.7% (0 h) to 13.6% (48 h) (*p* < 0.05) and the late cell apoptotic rate from 6.2% (0 h) to 8.95% (48 h) (*p* < 0.01). In BGC823 cells, *H. zeylanica*-E2 treatment significantly increased the total cell apoptotic rate from 3.05% (0 h) to 5.9% (48 h) (*p* < 0.05) and the early cell apoptotic rate from 2.1% (0 h) to 4.35% (48 h) (*p* < 0.01) (Figure 2B). Notably, BGC823 cells treated with *H. zeylanica*-E2 exhibited apoptosis in a time-dependent manner, especially in early-stage apoptosis, which accounted for a higher proportion of early-stage apoptosis in cells incubated for 48 h compared with 24 h.

### Effects of H. zeylanica-E2 on GC Cell Apoptosis by Activation of Caspase 3/7, Bcl-2, Bax, and Cleaved Poly (ADP-Ribose) Polymerase

**Figure 3:**
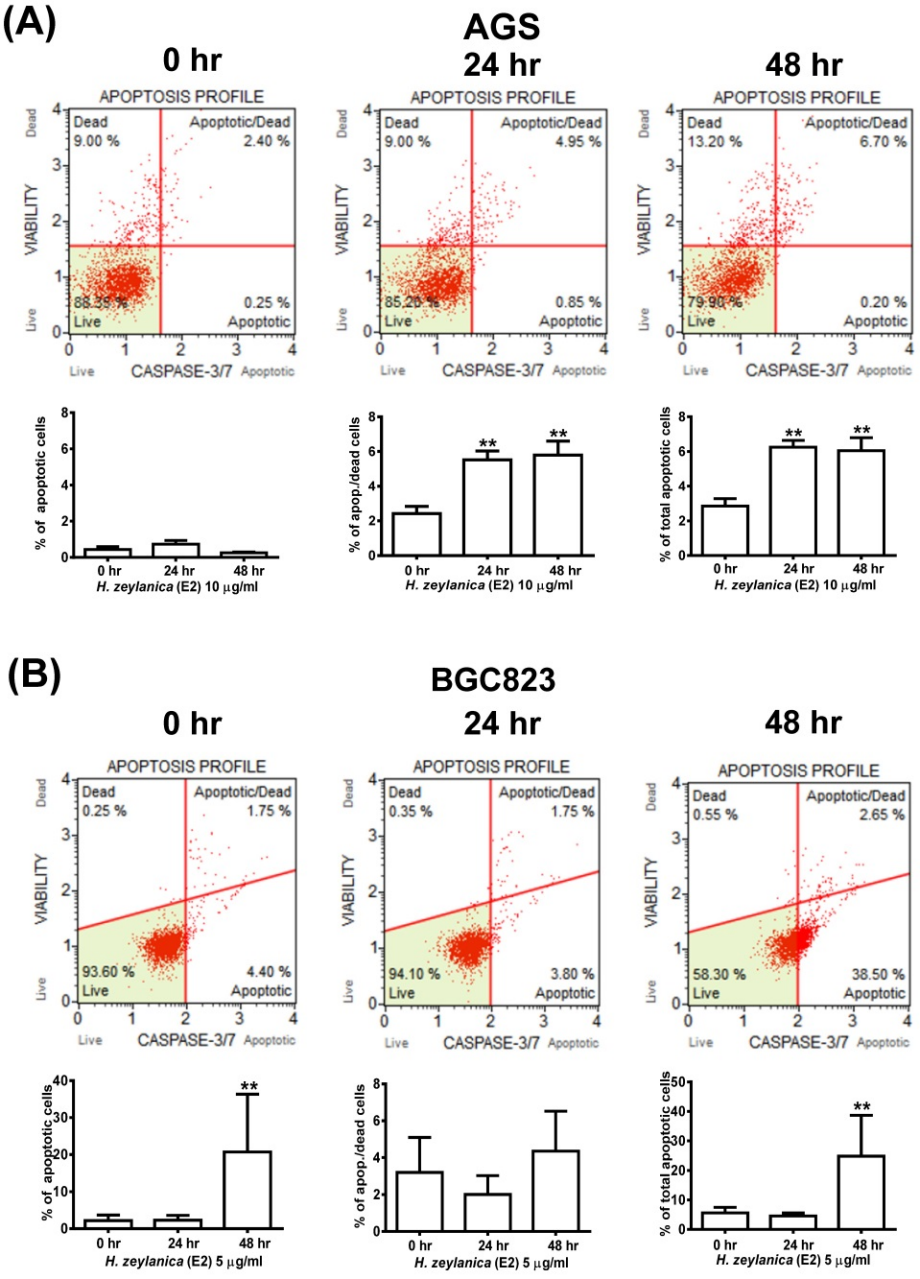
** Effects of *H. zeylanic*a-E2 on apoptosis of GC cells through changes in caspase 3/7 activity.** (**A**) AGS cells were treated with *H. zeylanica*-E2 (10 μg/mL), and (**B**) BGC823 cells were treated with *H. zeylanica*-E2 (5 μg/mL) for 0, 24, or 48 h. The cells from different experimental groups were collected and incubated with Muse™ Caspase-3/7 working solution (Merck) for 30 min at 37 °C and then mixed thoroughly with 7-AAD while protected from light at room temperature for 5 min and analyzed using a Muse™ Cell Analyzer (Merck). Four populations of cells (live, necrotic, apoptotic, and late apoptotic/dead) were quantified. The lower left shows live cells: caspase-3/7^-^ and 7-AAD^-^; lower right shows apoptotic cells exhibiting caspase-3/7 activity: caspase-3/7^+^ and 7-AAD^-^; upper left shows necrotic cells: caspase-3/7^-^ and 7-AAD^+^; and upper right shows late apoptotic/dead cells: caspase-3/7^+^ and 7-AAD^+^. Data are presented as mean ± SEM of at least three independent experiments. Student's-*t*-test or one-way ANOVA was used for comparison with the control. (**p < 0.01, *p < 0.05).

To explore further whether the activity of caspase 3/7 is involved in apoptosis, the cells were stained for caspases 3/7 and 7-AAD and then analyzed by flow cytometry. As shown in Figure 3A, GC cells treated with *H. zeylanica*-E2 for 0, 24, or 48 h exhibited apoptosis in a time-dependent manner. AGS cells exhibited a higher proportion of late-stage apoptosis, and BGC823 cells had a higher proportion of early-stage apoptosis. In AGS cells, *H. zeylanica*-E2 treatment significantly increased the total cell apoptotic rate from 2.65% at 0 h to 6.9% at 48 h (p < 0.01) and the late cell apoptotic rate from 2.4% at 0 h to 6.7% at 48 h (p < 0.01). In BGC823 cells, *H. zeylanica*-E2 treatment significantly increased the total cell apoptotic rate from 6.15% at 0 h to 41.15% at 48 h (p < 0.01) and the early cell apoptotic rate from 4.4% at 0 h to 38.5% at 48 h (p < 0.01) (Figure 3B).

**Figure 4:**
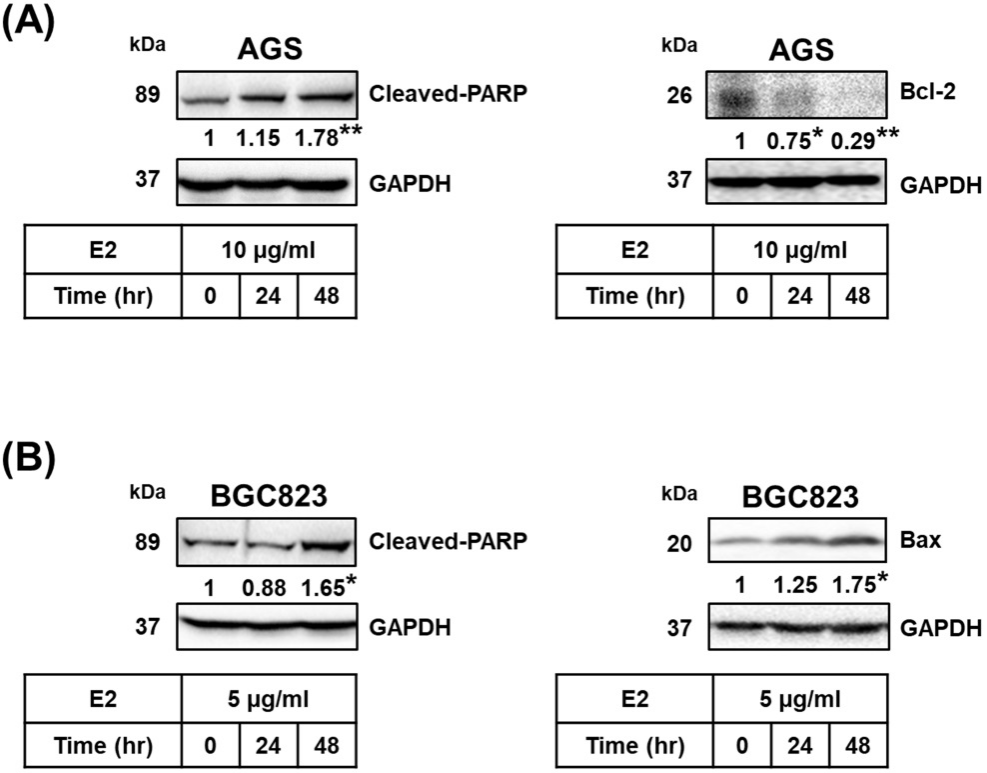
** Effects of *H. zeylanica*-E2 on apoptosis of GC cells through changes in Bcl-2, Bax, and cleaved PARP.** (**A**) AGS and (**B**) BGC823 cells were treated without or with *H. zeylanica*-E2 for 0, 24, or 48 h. The contents of proteins related to apoptosis (cleaved PARP, Bcl-2, and Bax) were quantified by Western blotting. GAPDH was used as the internal control, and the expression ratio of these proteins was quantified. Data are presented as mean ± SEM of three independent experiments. Student's-*t*-test or one-way ANOVA was used for comparison with the control. (*p < 0.05, **p < 0.01).

Proteins related to apoptosis were observed using Western blotting. Treatment with *H. zeylanica*-E2 for 24 or 48 h significantly increased cleaved poly (ADP-ribose) polymerase (PARP) content in AGS cells (by 1.78-fold at 48 h; *p* < 0.01) and BGC823 cells (by 1.65-fold at 48 h; *p* < 0.05) compared with the control (0 h). (Figure 4A and 4B, left). Treatment with *H. zeylanica*-E2 for 24 or 48 h significantly decreased Bcl-2 content in AGS cells (0.29 of the control value at 48 h; *p* < 0.01) and increased Bax content in BGC823 cells (by 1.75-fold at 48 h; *p* < 0.05) compared with the control (0 h) (Figure 4A and 4B, right). These data suggest that activated caspase 3/7, Bcl-2, Bax, and cleaved PARP may be involved in the effects of *H. zeylanica*-E2 on the endogenous proapoptotic pathway in GC cells.

### Effects of H. zeylanica-E2 on Apoptosis and Autophagy in GC Cells through changes in the Proinflammatory COX-2 Protein

**Figure 5:**
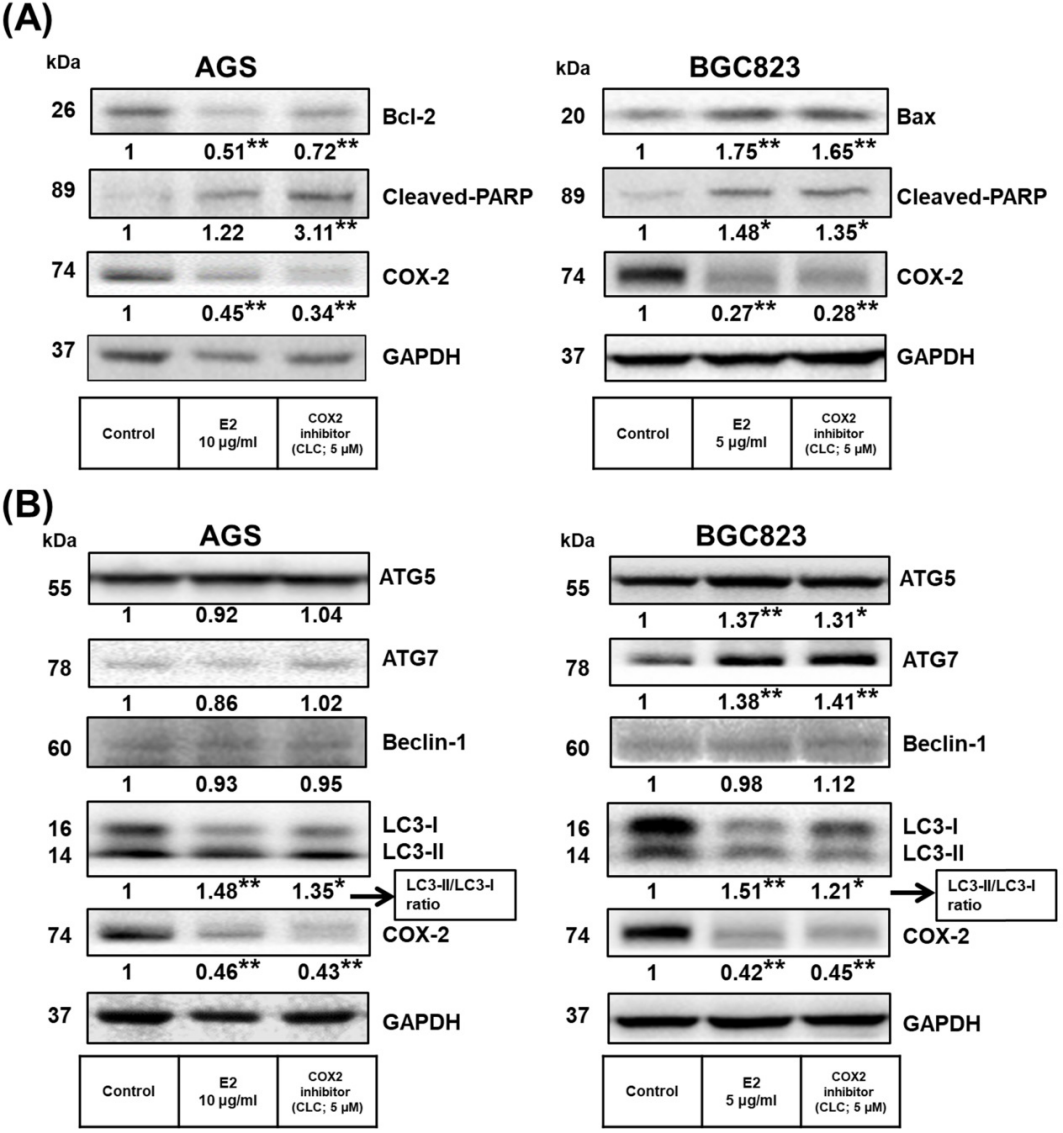
** Effects of *H. zeylanica*-E2 on GC cell apoptosis and autophagy via COX-2 protein.** (**A**) AGS and (**B**) BGC823 cells were treated without or with *H. zeylanica*-E2 and 5 μM CLC. The contents of proteins associated with apoptosis (cleaved PARP, Bcl-2, Bax, ATG5, ATG7, Beclin-1, LC3-I, LC3-II, and COX-2) were determined by Western blotting. GAPDH was used as an internal control. Autophagy is represented by LC3 conversion, expressed as the LC3-II/LC3-I ratio. Data are presented as mean ± SEM of three independent experiments. Student's-*t*-test or one-way ANOVA was used for comparison with the control. (*p < 0.05, **p < 0.01).

Previous studies found that COX-2 overexpression correlates with GC development 50-53. Additionally, *H. pylori* induces cell chronic inflammation, which leads to the aberrant *in vitro* expression of heat-shock protein 70 and apoptosis-related proteins (Bax and Bcl-2), and promotion of COX-2 expression in GC cells 54, 55. Next, we explored whether the inhibitory effects of *H. zeylanica*-E2 on proapoptotic proteins are associated with the proinflammatory COX-2 protein in GC cells. As shown in Figure 5A, compared with the control, treatment of AGS cells with *H. zeylanica*-E2 or CLC significantly reduced the levels of Bcl-2 (0.51 and 0.72 of the control value, respectively; *p* < 0.01 for both) and COX-2 (0.45 and 0.34 of the control value; *p* < 0.01 for both). Treatment of AGS cells with *H. zeylanica*-E2 or CLC increased the cleaved PARP level (by 1.22- and 3.11-fold, respectively; *p* < 0.01 for both). A similar pattern was also observed in BGC823 cells. In BGC823 cells, treatment of *H. zeylanica*-E2 or CLC significantly increased the levels of Bax (1.75- and 1.65-fold, respectively; *p* < 0.01 for both) and cleaved PARP (1.48- and 1.35-fold, respectively; *p* < 0.05 for both), but decreased the COX-2 level (to 0.27 and 0.28 of the control value, respectively; *p* < 0.01 for both). These data suggest that proinflammatory COX-2 protein is involved in GC cell apoptosis induced by the E2 extract.

Next, we examined whether *H. zeylanica*-E2 can protect cells against oxidative damage by modulating autophagy. The levels of ATGs in GC cells were measured using Western blotting. As shown in Figure 5B, *H. zeylanica*-E2 and CLC significantly decreased the accumulation of COX-2 proteins. By contrast, the ratios of the two forms of the microtubule-associated protein 1A/1B light chain 3 (LC3), LC3-II/LC3-I were significantly increased in AGS and BGC823 cells. Notably, in BGC823 cells, the expression of ATG5 was increased significantly (by 1.37- and 1.31-fold for E2 and CLC; *p* < 0.01, *p* < 0.05, respectively) and that of ATG7 was increased significantly (by 1.38- and 1.41-fold, respectively; *p* < 0.01 for both). Taken together, these findings suggest that apoptosis of GC cells caused by *H. zeylanica*-E2 involves effects on autophagy that are partly related to COX-2 activation.

### Effects of H. zeylanica-E2 on PGE_2_ production through downregulation of the Expression of Proinflammatory COX-2 in GC Cells

**Figure 6:**
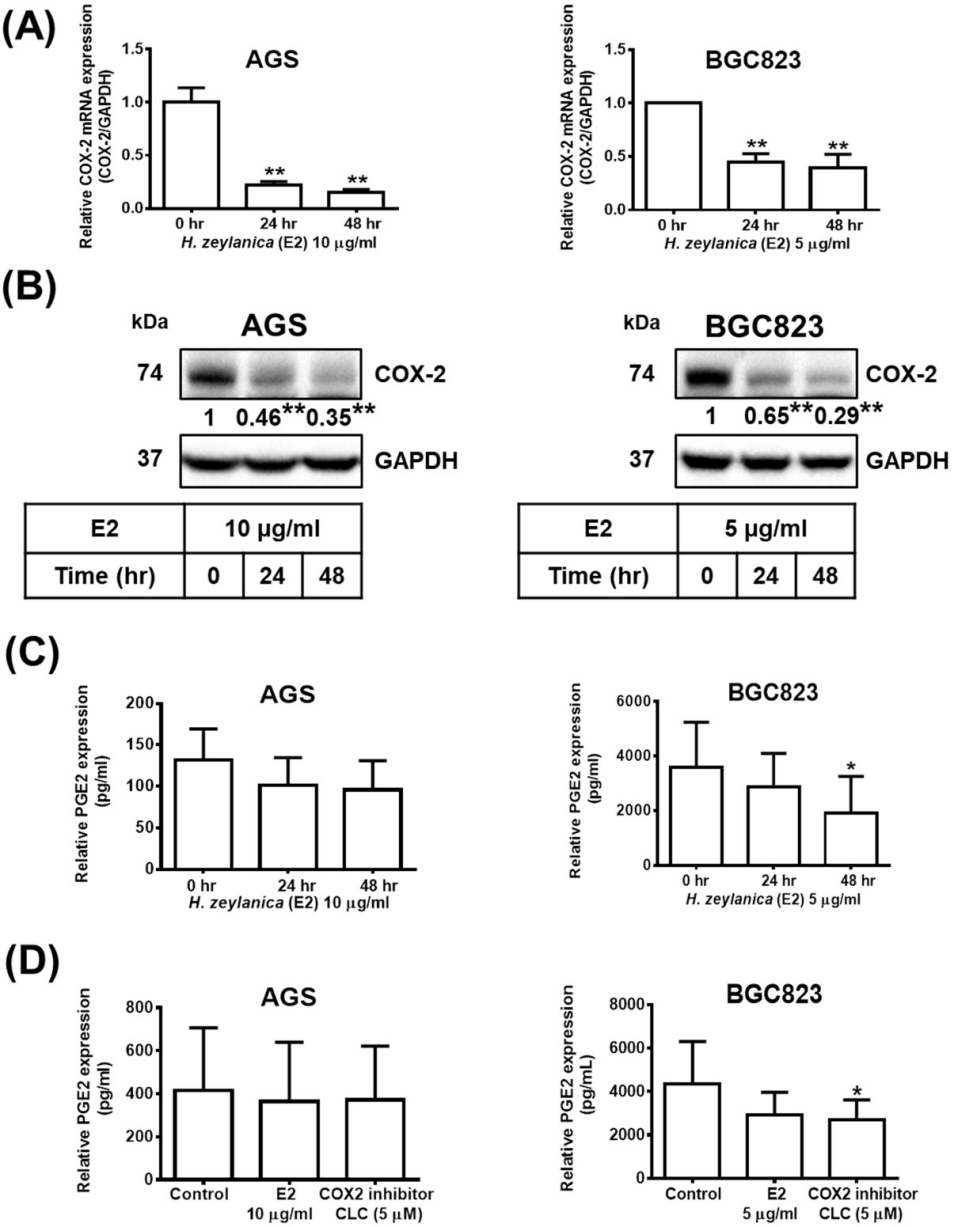
** Effects of *H. zeylani*ca-E2 on PGE_2_ expression mediated through COX-2 expression.** AGS cells were treated with 10 μg/mL *H. zeylanica*-E2, and BGC823 cells were treated with 5 μg/mL* H. zeylanica*-E2 for 0, 24, or 48 h. The *COX-2* mRNA transcript and protein expression levels were determined using (**A**) quantitative reverse transcription polymerase chain reaction (qRT-PCR) and (**B**) Western blotting. GAPDH was used as an internal control. (**C**) The PEG_2_ protein level was measured using an enzyme-linked immunosorbent assay (ELISA). (**D**) AGS and BGC823 cells were treated without or with *H. zeylanica*-E2 or 5 μM CLC for 24 h. The conditioned medium was collected and the PEG_2_ level was measured by ELISA. Data are presented as mean ± SEM of three independent experiments. Student's-*t*-test or one-way ANOVA was used for comparison with the control. (*p < 0.05, **p < 0.01).

Next, to determine whether the *in vitro* antitumor effects of *H. zeylanica* are mediated through proinflammatory COX-2, we investigate the molecular mechanism underlying the effects of the extract. Treatment with *H. zeylanica*-E2 significantly reduced the expression of *COX-2* mRNA and protein levels in AGS cells (0.17- and 0.35-fold at 48 h, respectively; *p* < 0.01 for both) and in BGC823 cells (0.38- and 0.29-fold at 48 h; *p* < 0.05 and *p* < 0.01, respectively) in a time-dependent manner (Figure 6A and 6B). It was found in GC cells that the longer the treatment time of *H. zeylanica*-E2, the less the expression of PGE_2_. Among them, only treatment with *H. zeylanica*-E2 for 48 h significantly reduced the PGE_2_ protein level in BGC823 cells compared with control cells (0 h) (Figure 6C). As shown in Figure 6D, PGE_2_ protein level did not change significantly in AGS cells treated with *H. zeylanica*-E2 or CLC (0.89 and 0.91 of the control values, respectively) compared with untreated control cells. In BGC823 cells, *H. zeylanica*-E2 and CLC treatment reduced PGE_2_ protein level (0.72 and 0.65 of the control values, respectively), but only the change induced by CLC was significant (*p* < 0.01) compared with untreated control cells. These data suggest that the *H. zeylanica*-E2-induced decrease in PGE_2_ production was mediated through proinflammatory COX-2 in GC cells.

### Effects of *H. zeylanica*-E2 on TNF-α Induced COX-2 Expression by modulating cPLA2

**Figure 7:**
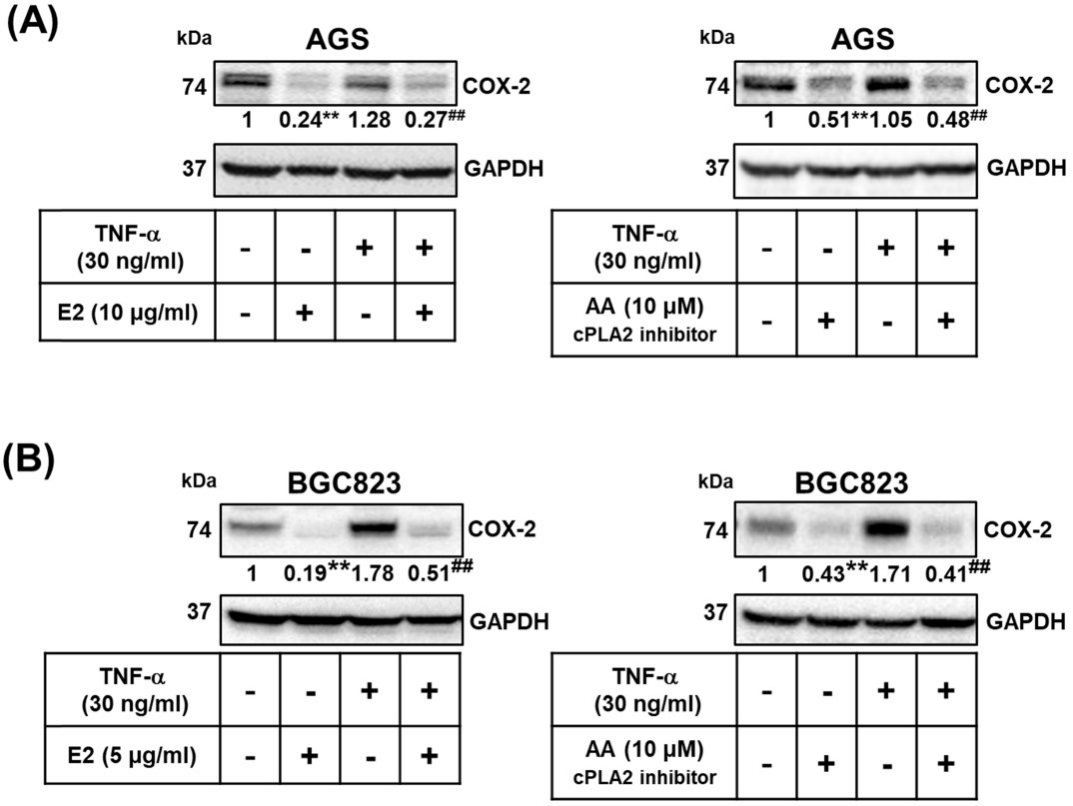
** Role of cPLA2 in the effects of *H. zeylanica*-E2 on TNF-α-induced COX-2 expression.** (**A**) AGS cells were pretreated with or without *H. zeylanica*-E2 (left) or AA (right) and then incubated with TNF‐α for 24 h. (**B**) BGC823 cells were pretreated with *H. zeylanica*-E2 (left) or AA (right) and then stimulated with TNF‐α for 24 h. The COX-2 protein level was determined by Western blotting. GAPDH was used as a loading control. The expression level of COX-2 was quantified according to the position of the electrophoresis band. The results are expressed as the quantified value for COX-2 divided by that of GAPDH. Data are presented as mean ± SEM of three independent experiments. Student's-*t*-test or one-way ANOVA was used for comparison with the control. (^#^*p* < 0.05, ^##^*p* < 0.01 compared with the TNF‐α-stimulated group; *p < 0.05, **p < 0.01 compared with the control).

We examined whether the effects of *H. zeylanica*-E2 involve the proinflammatory COX-2-mediated pathway. TNF-α is produced by tumor and immune cells, activates the COX‐2-PGE_2_ pathway, and plays an important role in the maintenance of tumor cells 51, 53, 56, 57. We examined whether *H. zeylanica*-E2 can also inhibit COX-2 expression in AGS and BGC823 cells treated with TNF-α. *H. zeylanica*-E2 significantly reduced the expression of COX-2 protein in AGS cells (0.24 of the control value; *p* < 0.01) and in BGC823 cells (0.19 of the control value; *p* < 0.01) compared with control cells. *H. zeylanica*-E2 also significantly reduced the expression of COX-2 protein in TNF‐α-stimulated AGS cells (0.27 of the control value; *p* < 0.01) and in BGC823 cells (0.51 of the control value; *p* < 0.01) compared with control cells stimulated with TNF‐α (Figure 7A, left and Figure 7B, left).

Next, to evaluate whether cPLA2 mediates COX-2 expression, AGS and BGC823 cells were pretreated with or without AA, and then incubated with or without TNF‐α. AA significantly reduced the expression of COX-2 protein in AGS cells (0.51 of the control value; *p* < 0.01) and in BGC823 cells (0.43 of the control value; *p* < 0.01) compared with control cells. AA also significantly reduced TNF‐α-stimulated COX-2 expression in AGS cells (0.48 of the control value; *p* < 0.01) and in BGC823 cells (0.41 of the control value; *p* < 0.01) compared with control cells stimulated with TNF‐α (Figure 7A, right and Figure 7B right). These findings suggest that *H. zeylanica*-E2 reduced the TNF-α-induced expression of proinflammatory COX-2 expression via cPLA2.

### Effects of *H. zeylanica*-E2 on GC Cell Proliferation and Colony Formation through Cell Cycle Arrest in G0/G1 and G2/M Phases

**Figure 8:**
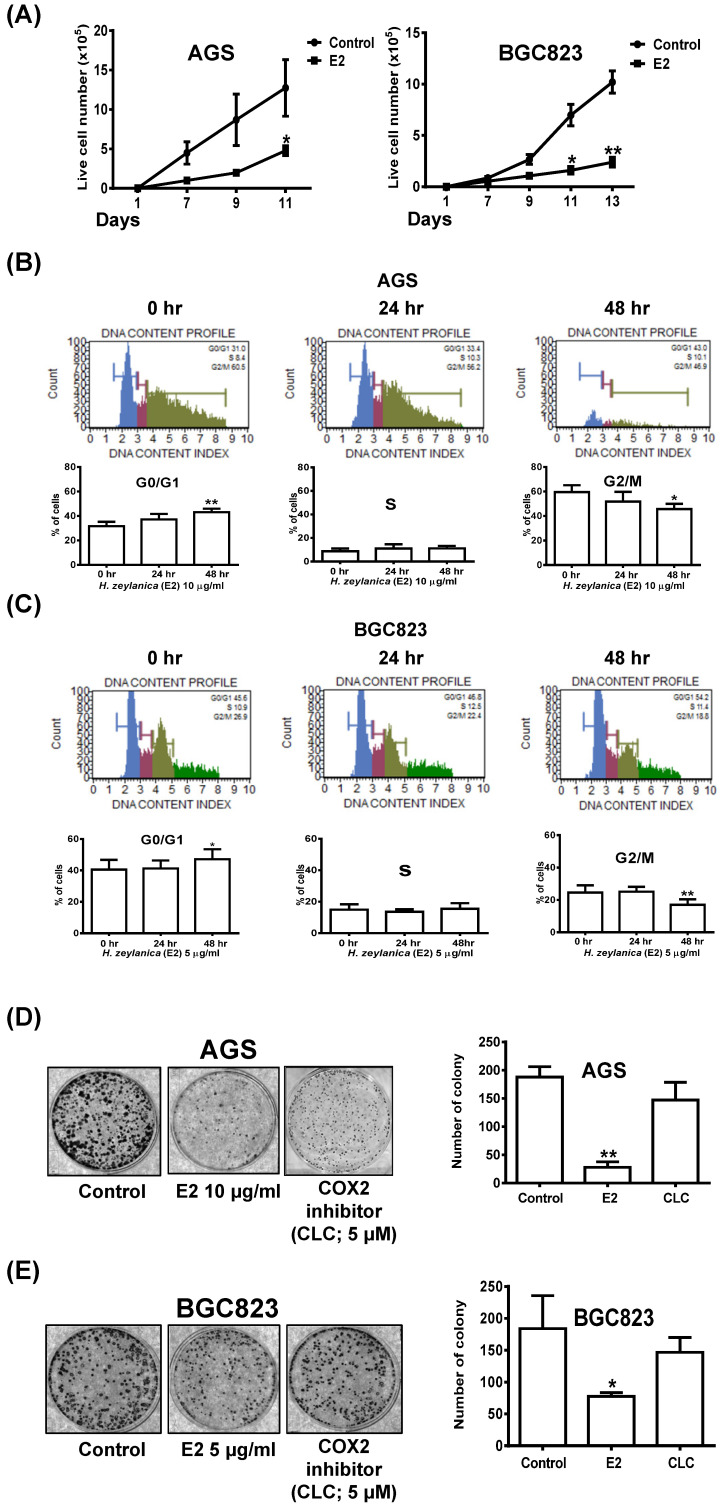
** Effects of H. zeylanica-E2 on GC cell proliferation, colony-forming ability, and cell cycle arrest in G0/G1 and G2/M phases.** (**A**) AGS and BGC823 cells were treated with H. zeylanica-E2 for various times in the colony-forming assay, and the number of cells was counted after staining with crystal violet. Each experimental group included five random fields for analysis. (**B**) AGS and (**C**) BGC823 cells were treated with H. zeylanica-E2 for 0, 24, or 48 h. The cells from different experimental groups were collected and incubated with Muse™ Cell Cycle Reagent (Merck) while protected from light at room temperature for 30 min. The cell populations in each phase of the cycle (G0/G1, S, and G2/M) were analyzed using a Muse™ Cell Analyzer (Merck). (**D**) AGS and (**E**) BGC823 cells were treated with H. zeylanica-E2 or 5 μM CLC for (**D**) 11 or (**E**) 13 days, the colony-forming assay was performed, and the number of cells was counted after staining with crystal violet. Each experimental group included five random fields for analysis. Data are presented as mean ± SEM of three independent experiments. Student's-t-test or one-way ANOVA was used for comparison with the control. (*p < 0.05, **p < 0.01).

To examine the effects of *H. zeylanica*-E2 on cell proliferation in AGS and BGC823 cells, we performed a cell proliferation assay. Cell growth curves showed that *H. zeylanica*-E2 significantly inhibited cell proliferation in AGS and BGC823 cells on day 11 (*p* < 0.05). This marked inhibition continued to day 13 in BGC823 cells (*p* < 0.01) (Figure 8A). *H. zeylanica*-E2 significantly inhibited GC cell proliferation in a time-dependent manner. These findings suggest that *H. zeylanica*-E2 stimulation has a significant antiproliferative effect in AGS and BGC823 cells.

Next, flow cytometry was performed to examine whether *H. zeylanica*-E2 affects cell cycle progression in GC cells. Compared with the control (G0/G1 phase: 31.6%; G2/M phase: 59.5%), in AGS cells, *H. zeylanica*-E2 treatment for 48 h significantly increased the percentage of cells in G0/G1 phase (43.1%; *p* < 0.01) and reduced the percentage of cells in G2/M phase (45.7%; *p* < 0.05). *H. zeylanica*-E2 significantly prevented cell cycle progression in AGS cells in a time-dependent manner (Figure 8B). Compared with the control (G0/G1 phase: 40.6%; G2/M phase: 24.5%), in BGC823 cells, *H. zeylanica*-E2 treatment for 48 h significantly increased the percentage of cells in G0/G1 phase to 47.2% (*p* < 0.05) and reduced the percentage of cells in G2/M phase (17%, *p* < 0.01) (Figure 8C). However, the percentages of cells in S phase did not change in AGS and BGC823 cells treated with *H. zeylanica*-E2 (Figure 8B, middle and Figure 8C, middle). These findings suggest that *H. zeylanica*-E2 treatment inhibited cell growth in GC cells through cell cycle arrest in G0/G1 and G2/M phases.

The entire progression of cancer cell metastasis requires the ability to enrich abilities for migration/invasion, survival, and colony formation 58. Next, we explored the effect of *H. zeylanica*-E2 on the colony formation of GC cells. As for the cell proliferation results presented above, *H. zeylanica*-E2 treatment significantly inhibited the colony-forming ability of AGS and BGC823 cells (0.15 and 0.42 of the control values; *p* < 0.01 and *p* < 0.05, respectively). We note that the colony-forming ability was not significantly inhibited by CLC in AGS and BGC823 cells (0.78-fold, NS; 0.80-fold, NS), respectively (Figures 8D and 8E). These findings suggest that the inhibitory effect of *H. zeylanica*-E2 on the colony-forming ability of GC cells does not involve COX-2.

### Possible Participation of COX-2 in *H. zeylanica*-E2-Reduced AGS Cell Mobility

**Figure 9:**
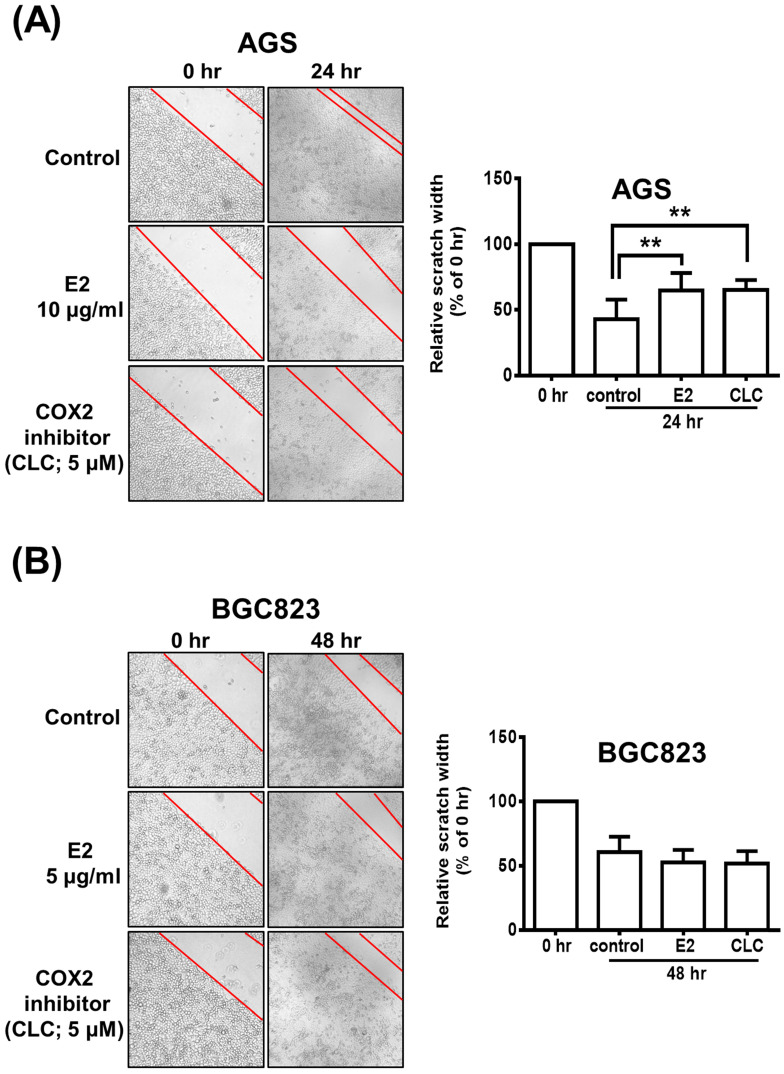
** The role of COX-2 in H. zeylanica-E2-reduced AGS cell mobility.** (**A**) Cell mobility was analyzed using the scratch wound-healing assay in (**A**) AGS cells treated with H. zeylanica-E2 or CLC for 24 h and (**B**) BGC823 cells treated with these drugs for 48 h. The control group was at 24 h or 48 h after scratch wound healing assay was induced and did not involve any drugs. Each experimental group included five random fields for analysis. Magnification, ×100. Data are expressed as the mean ± SEM of triplicate migration wells. Student's-t-test or one-way ANOVA was used for comparison with the control. (**p < 0.01).

The scratch wound-healing assay was performed to investigate the effects of *H. zeylanica*-E2 or CLC on the migration of AGS and BGC823 cells. AGS cells were treated with *H. zeylanica*-E2 or CLC 24 h after the scratch wound was induced. Compared with control cells, the wound healing rate was significantly 1.52-fold and 1.53-fold higher for E2 and CLC, respectively (both *p* < 0.01) (Figure 9A). However, compared with the control group, these drugs did not affect the wound healing rate in BGC823 cells (Figure 9B). These data support the hypothesis that *H. zeylanica*-E2 inhibits cell migration and that this effect may involve COX-2 protein in AGS cells, but not in BGC823 cells.

**Figure 10:**
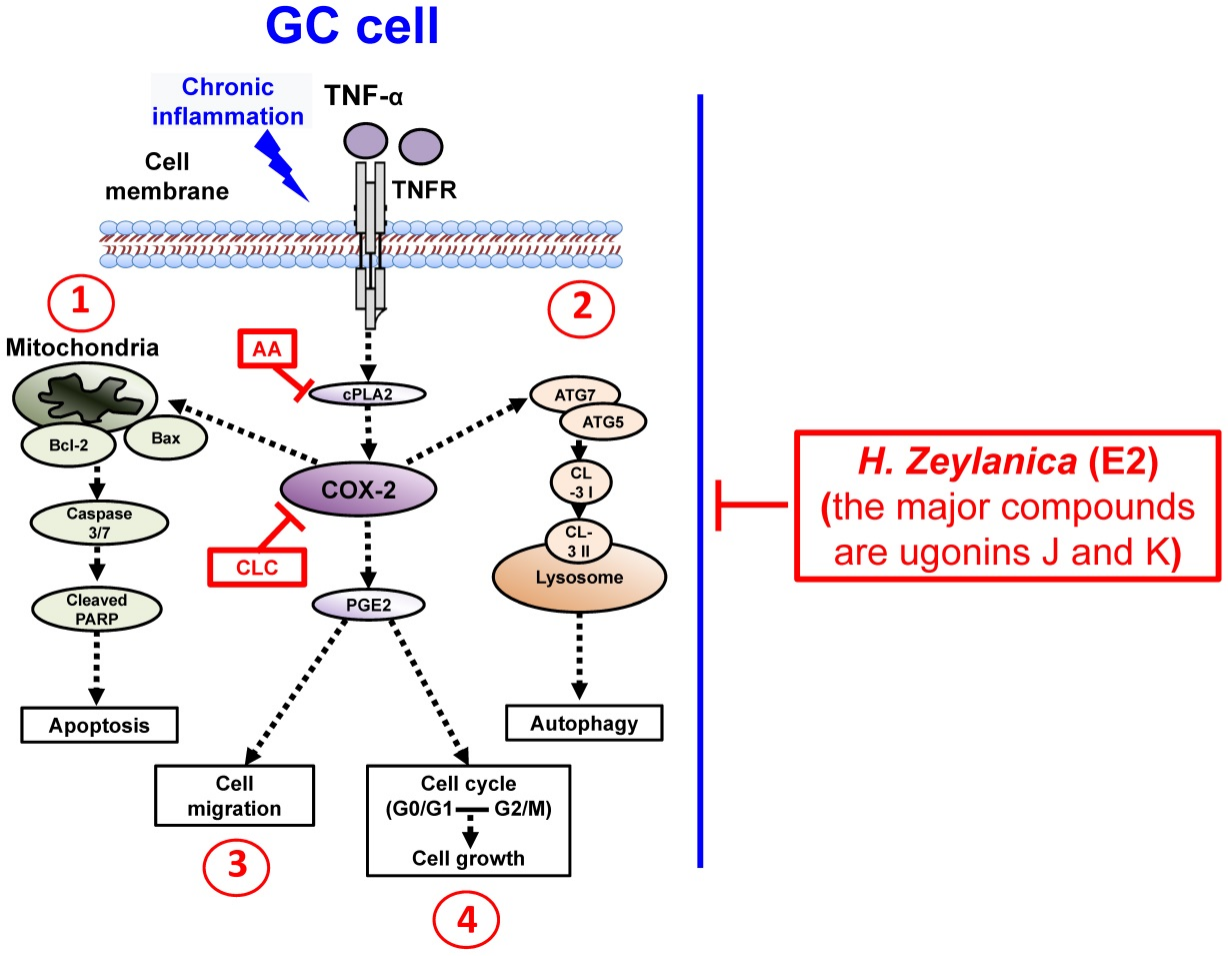
** Schematic diagram characterizing the anticancer mechanism underlying the effects of H. zeylanica-E2 treatment in GC cells.** H. zeylanica-E2 may increase GC apoptosis, autophagy, and cell cycle arrest, but may suppress GC migration. Apoptosis and autophagy are regulated by endogenous apoptosis-associated proteins (Bcl-2, Bax, caspase 3/7, and cleaved PARP), and autophagy-associated proteins (ATG7, ATG5, LC3-I, and LC3-II). Cell cycle arrest may occur in G0/G1 and G2/M. All of these anticancer effects may be partly ascribed to TNF-α activation of the proinflammatory COX-2 pathway. Taken together, these anticancer effects suggest that H. zeylanica-E2 has potential as a chemotherapeutic drug for the treatment of GC.

To summarize our findings, we identified four *in vitro* antitumor activities of *H. zeylanica*-E2 in GC cells. *H. zeylanica*-E2 increased GC apoptosis by modulating endogenous apoptosis-associated proteins (Bcl-2, Bax, caspase 3/7, cleaved PARP) and increased GC autophagy by regulating autophagy-associated proteins (ATG7, ATG5, LC3-I, and LC3-II). *H. zeylanica*-E2 inhibited GC cell cycle progression, which led to cell cycle arrest in G0/G1 and G2/M and suppressed GC migration. These findings lead us to conclude that the anticancer effects of *H. zeylanica*-E2 in GC cells can be partly ascribed to TNF-α activation of the proinflammatory cPLA2-COX-2-PGE_2_ pathway. We propose that *H. zeylanica*-E2 has potential as a drug for adjunctive therapy in the treatment of GC (Figure 10).

## Discussion

Chronic inflammation is considered a vital factor involved in the development and progression of GC 19. Masami *et al.* have reported that *H. pylori* infection of GC cells activates the NF-κB pathway, which stimulates the expression of TNF-α and inflammatory proteins such as COX-2 and PGE_2_ 23, 24, 59. *H. pylori* infection is also related to the occurrence and progression of GC 60, 61. In general, TNF-α in serum plays an important role in tumor necrosis and defense against infectious diseases. However, overexpression of TNF-α may induce a strong inflammatory response and organ failure, which can increase GC risk 60, 61.

Recent studies have reported that COX-2 is overexpressed in several cancers, such as colorectal cancer, HCC, GC, pancreatic cancer, and breast cancer 62-66. Overexpression of COX-2 and MMP-13 has been reported in many clinical GC specimens and may be related to tumor invasion, metastasis, tumor-node-metastasis stage, and overall survival of patients with GC. It has been suggested that COX-2 and MMP-13 may be biomarkers of GC progress 50. Several recent studies have also reported that upregulation of COX-2 is involved in the development of *Hp*-induced inflammation, which is involved in the progression from precancerous lesions to GC. COX-2 also promotes cell proliferation, angiogenesis, and anti-apoptosis in GC cells 24, 64, 67. Romano *et al*. have shown that *H. pylori* causes the accumulation of COX-2 and PGE_2_ in GC 68. Another group has also reported similar results. COX-2 overexpression leads to the accumulation of PGE_2_ in GC cells, where it becomes a key factor in maintaining tumor survival 69. The public NCBI web GEO profile (ID: GDS1210/U04636) indicates that COX-2 expression is higher in GC tissues (T = 22; mean = 36.44) than in normal control tissues (N = 8; mean = 8.69) (*p <* 0.0011, unpaired *t*-test). The precise roles of COX-2 in GC require further investigation.

Traditional COX inhibitors such as nonsteroidal anti-inflammatory drugs (NSAIDs) can reduce the risk of GC 70, 71. However, NSAIDs also have side effects such as gastric bleeding or gastric perforation because of the simultaneous inhibition of COX-1 and downstream PGE_2_, which lead to alterations in the gastric mucosa and may cause injury to the stomach wall 72, 73. Therefore, it is important to develop specific COX-2 inhibitors that do not affect the performance of COX-1. COX-2-specific inhibitors include CLC and rofecoxib. anti-inflammatory COX-2 inhibitors prevent the production of COX-2 enzyme, which can cause stomach irritation and pain. Anti-inflammatory COX-2 inhibitors prevent the production of COX-2 but do not affect the production of COX-1. COX-1 protects the stomach wall 74, 75. Traditional analgesics cannot distinguish between COX-1 and COX-2, and their use can cause stomach ulcers or bleeding. However, the use of COX-2-specific inhibitors has some contraindications. Because COX-2-specific inhibitors cannot block the production of systemic prostacyclin or inhibit thromboxane A2, these drugs may alter the balance between prothrombotic and antithrombotic activities *in vivo*, which may increase the risk of adverse cardiovascular events such as myocardial infarction. The European Union is amending the labeling instructions for anti-inflammatory COX-2 inhibitor drugs to strengthen the warning that these drugs can cause gastrointestinal and increase cardiovascular risk 76-78.

*H. zeylanica* has marked extensive pharmacological effects, such as hepatoprotective, anti-inflammation, antioxidation, antiosteoporosis, immunomodulatory, and neuroprotective activities 30-32, 36. Notably, *H. zeylanica* exhibits striking anticancer activity in various human *cancers* 79, 80. However, the mechanisms responsible for these *H. zeylanica* anticancer actions remain unclear for GC. Therefore, we focused on evaluating whether *H. zeylanica* has anticancer effects in GC cells. Its inflammation-inhibiting effect has been confirmed repeatedly in animal experiments, and its specific anticancer mechanisms have received much attention 81, 82. However, there are concerns about the safety, efficacy, and the absence of reliable clinical research. Additionally, its potential medicinal properties remain unconfirmed because its use in oral therapy requires further investigation.

We analyzed *H. zeylanica* extracts (E1 and E2) using HPLC. The *H. zeylanica* secondary extracts from *H. zeylanica*-E1 and *H. zeylanica*-E2 were prepared, and we found that *H. zeylanica* contained mostly flavonoids compounds (the major compounds are ugonins J and K) 30-33. We found that incubation of cells for 24 or 48 h with *H. zeylanica*-E1 at doses ≤1000 μg/mL did not significantly affect the viability of the two GC cell lines (AGS and BGC823) compared with normal GES-1 cells. Interestingly, *H. zeylanica*-E2 exhibited dose- and time-dependent effects on apoptosis in GC cells. We also found that *H. zeylanica*-E2 was selectively toxic to GC cells but had minimal or no toxicity to normal GES-1 cells. Liou *et al.* reported that *H. zeylanica*-E1 and* H. zeylanica*-E2 contain different compounds (two major compounds, ugonins J and K) as analyzed by HPLC 39. In future studies, we plan to identify, isolate, and purify the bioactive compounds in *H. zeylanica*-E2 that exhibit anticancer activity induced by TNF‐α in GC cells.

Our experimental data show the *in vitro* antitumor effects of *H. zeylanica-E2* in GC cells. We found several main effects of *H. zeylanica-*E2, First, this extract increased GC apoptosis by modulating endogenous apoptosis-associated proteins such as Bcl-2, caspase 3/7, and cleaved PARP in AGS cells. This effect was time dependent, especially in late-stage apoptosis. *H. zeylanica-*E2 increased GC apoptosis by modulating endogenous apoptosis-associated proteins such as Bax, caspase 3/7, and cleaved PARP proteins in BGC823 cells in a time-dependent manner, especially in late-stage apoptosis. Second, *H. zeylanica-E2* increased GC autophagy by regulating autophagy-associated proteins and increasing the LC3-II/LC3-I ratio. *H. zeylanica*-E2 and CLC significantly decreased the accumulation of COX-2 protein in AGS cells. Notably, the LC3-II/LC3-I ratio and ATG5 and ATG7 expression were significantly increased in BGC823 cells. CLC significantly decreased the accumulation of COX-2 protein in BGC823 cells. Third, *H. zeylanica*-E2 significantly inhibited GC cell cycle progression in a time-dependent manner, which led to cell cycle arrest in G0/G1 and G2/M in AGS and BGC823 cells. Fourth,* H. zeylanica*-E2 inhibited cell migration via COX-2 protein in AGS cells but not in BGC823 cells.

Differentiated GC cells form aggregated masses and colonies, which suggests that cell mobility is specific to undifferentiated GC cells. However, different two-type reactions between AGS cells and BGC823 cells with pro-apoptosis, pro-autophagy, and antimigration through different proteins interact. We suggest that the source and degree of differentiation may affect GC cells differently. Histomorphologically, GC is divided into two main types: intestinal-differentiated and diffuse-undifferentiated 83, 84. However, lesions with a similar type may differ in their biological aggressiveness and response to therapy. The molecular events related to the development and progression of GC also seem to differ 84. The involvement of COX-2 activity in the tumorigenic microenvironment is poorly understood. Data from our current study provide new insights that will help to clarify the mechanisms underlying the regulation of GC tumorigenesis by COX-2. Future extensive prospective studies are warranted before COX-2 can be clinically applied as a therapeutic agent for the treatment of GC.

## Conclusions

Our results demonstrated that E2, but not E1, significantly increased GC apoptosis, autophagy, and cell cycle arrest, but may suppress GC migration. Apoptosis and autophagy are regulated by endogenous apoptosis-associated proteins (Bcl-2, Bax, caspase 3/7, and cleaved PARP), and autophagy-associated proteins (ATG7, ATG5, LC3-I, and LC3-II). Cell cycle arrest may occur in G0/G1 and G2/M. All of these anticancer effects may be partly ascribed to the TNF-α-activated proinflammatory cPLA2-COX-2-PGE_2_ pathway. Taken together, these anticancer effects suggest that *H. zeylanica-*E2 has potential as a novel adjunctive agent for the treatment of GC.

## Supplementary Material

Supplementary figure.
