# Supplementary material for: Anticancer Effects of Helminthostachys zeylanica Ethyl acetate Extracts on Human Gastric Cancer Cells through Downregulation of the TNF-α-activated COX-2-cPLA2-PGE2 Pathway
Source: J Cancer. 2021 Oct 17;12(23):7052–68. doi: 10.7150/jca.64638 (PMC8558661; doi:10.7150/jca.64638)
Supplement: Supplementary file 1 — Supplementary figure. [file jcav12p7052s1.pdf]

Anticancer Effects of *Helminthostachys zeylanica* Ethyl acetate Extracts on Human Gastric Cancer Cells Through Downregulation of the TNF-α-activated COX-2–cPLA2–PGE_2 Pathway

Ming-Ming Tsai [1,†], Horng-Chyuan Lin [2,†], Ming-Chin Yu [3], Wan-Jung Lin [4], Mei-Yi Chu [4], Ching-Ching Tsai [5] and Ching-Yi Cheng [4,6,*]

1 Department of Nursing, Division of Basic Medical Sciences, Research Center for Chinese Herbal Medicine, College of Human Ecology, Chang Gung University of Science and Technology, Taoyuan, Taiwan and Department of General Surgery, Chang Gung Memorial Hospital at Chiayi, Chiayi, Taiwan; mmtsai@mail.cgust.edu.tw (M.M.T.)

2 Department of Thoracic Medicine, Chang Gung Memorial Hospital at Linkou and College of Medicine, Chang Gung University, Taoyuan, Taiwan; lin53424@gmail.com (H.C.L.)

3 Department of Surgery, New Taipei Municipal TuCheng Hospital, Chang Gung Memorial Hospital at Linkou, and College of Medicine, Chang Gung University, Taoyuan, Taiwan; a75159@cgmh.org.tw (M.C.Y.)

4 Graduate Institute of Health Industry Technology, Research Center for Chinese Herbal Medicine and Research Center for Food and Cosmetic Safety, College of Human Ecology, Chang Gung University of Science and Technology, Taoyuan, Taiwan; gop6000000@gmail.com (W.J.L.); mychu@mail.cgust.edu.tw (M.Y.C.); jennycheng@mail.cgust.edu.tw (C.Y.C.)

5 Department of Nursing, College of Nursing, Chang Gung University of Science and Technology, and Department of Cardiology, Chang Gung Memorial Hospital at Linkou, Taoyuan, Taiwan; cctsai@mail.cgust.edu.tw (C.C.T.)

6 Department of Pulmonary Infection and Immunology, Chang Gung Memorial Hospital at Linkou, Taoyuan, Taiwan (C.Y.C.)

* Correspondence: jennycheng@mail.cgust.edu.tw (C.Y.C.); Telephone: 886-3-2118999 EXT. 5114/Fax: 886-3-2118866

† These authors have contributed equally to this work.

Supplementary Data-Original data Figs 4-7 of WB

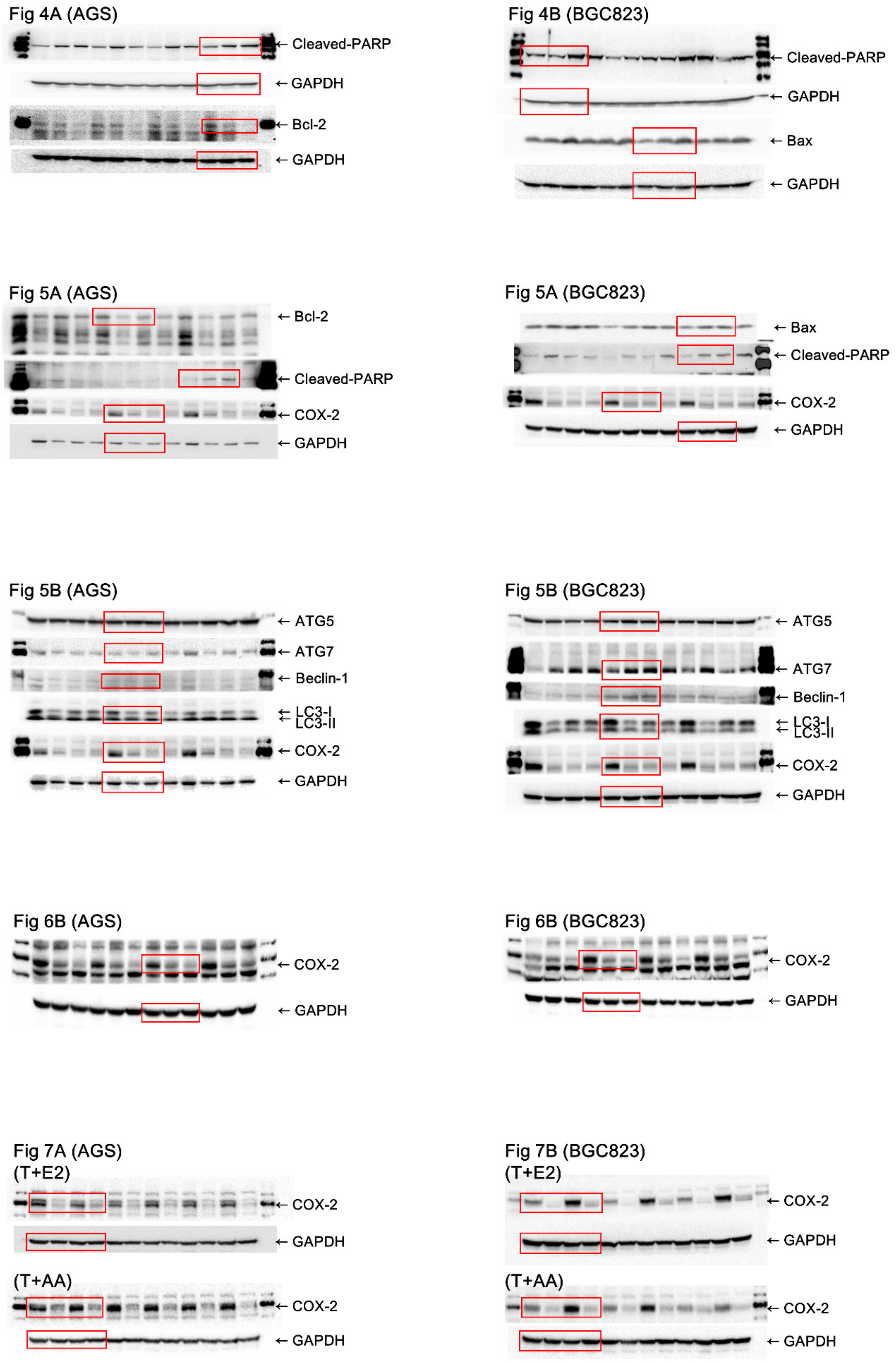

Fig SF1: The original data of figures 4-7. The effect of *H. zeylanica*-E2 on the protein expression in GC cells was determined by Western blotting. GAPDH was used as the internal control.
